# Microbiome diversity in soils of the agro-ecological zones of Bangladesh

**DOI:** 10.1128/mra.01237-24

**Published:** 2025-02-25

**Authors:** Nurul Islam, M. Nazmul Hoque, Sabbir Hossen, Abu Ahsan Gilman, Tofazzal Islam

**Affiliations:** 1 Soil Resource Development Institute Divisional Office, Rajshahi, Bangladesh; 2 Department of Gynecology, Obstetrics and Reproductive Health, Molecular Biology and Bioinformatics Laboratory, Bangabandhu Sheikh Mujibur Rahman Agricultural University (BSMRAU) 198780 , Gazipur, Bangladesh; 3 Crop Zoning Project of the Bangladesh Agricultural Research Council, Krishi Khamar Sarak, Farmgate 130054 , Dhaka, Bangladesh; 4 Institute of Biotechnology and Genetic Engineering (IBGE) BSMRAU 198780 , Gazipur, Bangladesh; California State University San Marcos, San Marcos, California, USA

**Keywords:** agro ecological zones, Soil microbiome, Soil bacteriome, Microbial diversity, Bangladesh soil

## Abstract

Diversity and composition of soil bacteriome across various agro-ecological zones (AEZs) in Bangladesh were unveiled using 16S rRNA gene sequencing. The mapping of bacterial communities in each AEZ has laid a foundation for detailed mapping and engineering of soil microbiome in deltaic Bangladesh soils for mitigating impact of climate change and promoting sustainable agriculture.

## ANNOUNCEMENT

Soil microbiome plays an important role in maintaining soil health, plant productivity, and soil ecosystem services (1, 2). Bangladesh heavily relies on intensive agriculture for food security, rural development, and poverty alleviation (3). Despite developing soil maps for all AEZs based on physico-chemical properties, the microbiome, key to nutrient cycling, soil productivity, and greenhouse gas dynamics, remains largely overlooked. Better understanding of microbial diversity across 30 AEZs in Bangladesh is critical for adopting strategies for sustainable agriculture and mitigation of climate change (4). Probiotic roles of soil microbiomes in enhancing crop productivity with minimal reliance on chemical inputs are well established (5, 6). However, utilizing these microbes for climate-resilient, sustainable agriculture in Bangladesh remains underexplored. This study investigates bacteriome diversity across 14 AEZs using 16S rRNA-gene amplicon sequencing.

The soil samples were collected from 14 AEZs of Bangladesh (Table 1) during March to August 2024. The sampling sites, located about 30–40 km apart, spanned coordinates 22.17–25.879° N and 88.25–92.18° E, characterized by a tropical wet and dry climate. To analyze bacterial communities across AEZs, composite soil samples were prepared by pooling three soil samples from each zone. Soil was sampled at a depth of 0–5 cm using a 2.5 cm diameter soil auger, yielding approximately 10 g of soil from each AEZ in an aseptic manner (7). Each sample was homogenized, processed, and sieved with a 6.35 mm mesh. DNA was extracted using the Qiagen DNeasy PowerSoil Kit (8). Amplicon libraries were prepared using the Nextera XT Index Kit (Illumina Inc.), and the V3–V4 regions were amplified through a 30-cycle PCR with 341F and 806R primers (7, 9) under conditions of 98°C denaturation, 50°C annealing, and 72°C extension, followed by a final extension at 72°C (10). Sequencing was conducted on the Illumina MiSeq platform using the MiSeq Reagent Kit v3, which employed 2 × 300 bp paired-end sequencing chemistry. FastQC v0.11.9 (11) was used to check the quality of the reads, while Trimmomatic v0.39 (12) removed Illumina adapters, known Illumina artifacts, and phiX reads. The demultiplexed sequencing data were processed using QIIME 2 (2023.2.0) and associated plugins (13). Sequences showing ≥98% similarity were grouped into operational taxonomic units (OTUs) using the SILVA database v.138 (14). Default parameters were used for bioinformatic analyses except where otherwise stated.

**TABLE 1:** Study sample information, Sequence Read Archive (SRA) accession numbers of the 16S rRNA amplicon sequences, and OTUs (operational taxonomic units) mapped against bacterial taxa in the soil samples from different agro-ecological zones (AEZs) of Bangladesh

Sample ID	Location	Coordinate	Source	pH	Soil organic matter	Soil texture	No. of raw reads	No. of mapped reads	No. of observed OTUs	No. of genera detected	SRA accession
AEZ-2	Narrow belts within and adjoining the channels of rivers in Nilphamari, Rangpur, Lalmonirhat, Kurigram, and Gaibandha district.	25.879° N, 88.257° E	Soil	Acidic (4.6)	Low (1.21)	Loam	446,896	26,303	363	227	SRR31219345
AEZ-4	Eastern half of Bogra district and most of Sirajganj district.	24.671° N, 89.546° E	Soil	Acidic (5.1)	Medium (2.82)	Silt loam	810,184	49,730	444	290	SRR31219339
AEZ-5	Most of this region lies in Noagaon and Narore districts. Small areas extend into Rajshahi, Bogra, and Sirajganj districts.	24.529° N, 89.175° E	Soil	Acidic (5.3)	Medium (2.69)	Silt loam	487,106	33,056	364	239	SRR31219334
AEZ-10	The region extends along the Ganges and lower Meghna river channels from the Indian border Nowabganj and Rajshahi District to the mouth of the Meghna estuary in Lakshmipur and Barisal district.	23.746° N, 89.769° E	Soil	Alkaline (7.1)	Low (1.88)	Silt clay loam	367,152	19,691	360	237	SRR31219333
AEZ-12	Nature, Pabna, Goalanda, Faridpur, Madaripur, Gopalganj and Sariatpur, eastern parts of Kushtia, Magura and Narial, north-eastern parts of Khulna and Bagerhat, northern Barisal, and southwestern parts of Manikganj, Dhaka, and Munshiganj districts.	23.572°N, 89.825° E	Soil	Acidic (6.9)	Medium (2.08)	Clay	574,064	20,790	305	210	SRR31219332
AEZ-13	All or most of Barisal, Jhalkati, Pirojpur, Patuakhali, Bagerhat, Barhuna, Khulna, and Satkhira districts. It includes the Khulna and Bagerhat Sundarbans Reserve Forests.	22.698° N, 89.518° E	Soil	Alkaline (7.2)	Low (1.08)	Silt loam	530,834	20,114	339	227	SRR31219344
AEZ-16	Parts of Kishoreganj, Brahmanbaria, Comilla, Chandpur, Narsingdi, and Narayanganj.	23.577° N, 90.788° E	Soil	Acidic (5.8)	Low (1.48)	Loam	508,112	29,252	398	262	SRR31219343
AEZ-17	Chandpur, Lakshimpur, and Noakhali.	23.120° N, 90.758° E	Soil	Acidic (5.7)	Medium (2.15)	Silt loam	255,788	15,144	304	202	SRR31219342
AEZ-19	Kishoreganj, Habiganj, Brahmanbaria, Comilla, Chadpur, Feni, Noakhali, Laksmipur, Narsingdi, Narayanganj, Dhaka, Shariatpur, Madaripur, Gopalganj, and Barisal.	23.185°N, 89.810° E	Soil	Acidic (6.6)	Medium (2.89)	Clay loam	341,424	16,440	359	235	SRR31219341
AEZ-25	Dinajpur, Gaibandha, Joypurhat, Bogra, Naogaon, Sirajganj, and Natore.	24.656° N, 89.323° E	Soil	Acidic (5.7)	Low (1.82)	Clay loam	528,176	27,085	374	252	SRR31219340
AEZ-26	Rajshahi, Nawabganj, and Naogaon.	24.849° N, 88.440° E	Soil	Acidic (6.0)	Low (1.86)	Clay loam	570,734	35,620	445	280	SRR31219338
AEZ-27	Dinajpur, Rangpur, Gaibandha, Joypurhat, and Bogra.	25.616° N, 89.190° E	Soil	Acidic (5.3)	Low (1.08)	Silt loam	342,928	16,257	305	208	SRR31219337
AEZ-28	Dhaka, Gazipur, Narsingdi, Narayanganj, Tangail, Mymensingh, and Kishoreganj.	24.665° N, 90.030° E	Soil	Acidic (3.4)	Medium (2.29)	Sandy loam	819,726	14,447	208	125	SRR31219336
AEZ-29	Khagrachhari, Chittagong Hill Tracts, Bandarban, Chittagong, Cox’s Bazar, Habiganj, and Moulivibazar.	22.170° N, 92.189° E	Soil	Acidic (4.1)	Medium (2.35)	Loam	616,240	19,237	226	149	SRR31219335

From 14 composite soil samples, 7,199,364 raw reads were obtained, averaging 514,240 per sample. After quality filtering, 343,166 reads (4.77%) were assigned to 544 bacterial OTUs (Table 1). Using the SILVA v.138 database, these 544 OTUs were taxonomically assigned to 16 bacterial phyla, 45 classes, 77 orders, 151 families, and 341 genera. The soil sample from AEZ-4 exhibited the highest number of OTUs, while the lowest number of OTUs was observed in AEZ-28 (Table 1). Across all AEZs, *Gemmata* (12.11%) was identified as the top abundant genus followed by *Planctomyces* (8.83%), *Nocardioides* (6.11%), *Anaerolinea* (5.28%), *Candidatus* (4.80%), *Solibacter* (4.34%), *Bacillus* (3.72%), *Clostridium* (3.60%), *Rhodoplanes* (3.51%), and *Pirellula* (3.46%). The rest of the bacterial genera had <3.0% relative abundances. This amplicon sequencing of AEZ soil bacteriomes provides a foundation for studying soil microbiome functions, fertility, productivity, and greenhouse gas dynamics to support sustainable crop production in Bangladesh.

## Data Availability

The 16S rRNA gene amplicon sequencing data are available at the NCBI Sequence Read Archive (SRA) under BioProject accession number PRJNA1181648. The accession numbers for all SRA experiments are listed in Table 1.
